# Evaluation of an evidence-based guidance on the reduction of physical restraints in nursing homes: a cluster-randomised controlled trial [ISRCTN34974819]

**DOI:** 10.1186/1471-2318-9-42

**Published:** 2009-09-07

**Authors:** Antonie Haut, Sascha Köpke, Anja Gerlach, Ingrid Mühlhauser, Burkhard Haastert, Gabriele Meyer

**Affiliations:** 1 University of Witten/Herdecke, Faculty for Medicine, Institute of Nursing Science, Stockumer Str. 12, 58453 Witten, Germany; 2 University of Hamburg, Unit of Health Sciences and Education, Martin-Luther-King-Platz 6, 20146 Hamburg, Germany; 3 MediStatistica, Lambertusweg 1b, 58809 Neuenrade, Germany

## Abstract

**Background:**

Physical restraints are regularly applied in German nursing homes. Their frequency varies substantially between centres. Beneficial effects of physical restraints have not been proven, however, observational studies and case reports suggest various adverse effects. We developed an evidence-based guidance on this topic. The present study evaluates the clinical efficacy and safety of an intervention programme based on this guidance aimed to reduce physical restraints and minimise centre variations.

**Methods/Design:**

Cluster-randomised controlled trial with nursing homes randomised either to the intervention group or to the control group with standard information. The intervention comprises a structured information programme for nursing staff, information materials for legal guardians and residents' relatives and a one-day training workshop for nominated nurses. A total of 36 nursing home clusters including approximately 3000 residents will be recruited. Each cluster has to fulfil the inclusion criteria of at least 20% prevalence of physical restraints at baseline. The primary endpoint is the number of residents with at least one physical restraint at six months. Secondary outcome measures are the number of falls and fall-related fractures.

**Discussion:**

If successful, the intervention should be implemented throughout Germany. In case the intervention does not succeed, a three-month pre-post-study with an optimised intervention programme within the control group will follow the randomised trial.

**Trial registration:**

ISRCTN34974819

## Background

The use of physical restraints with older people has been reported as common practice in numerous countries [1]. International studies documented prevalence rates between 2% and 70% [2,3]. Our epidemiological study in 30 German nursing homes with approximately 2400 residents confirms that physical restraints are applied as routine care measure as indicated by a prevalence of 26% (95% confidence interval 21 to 31). Centre differences were pronounced with a range of 4% to 59% [4]. A recent study comparing prevalences between five different countries confirms our findings [5].

The use of physical restraints is widely justified by nurses as safety measure, primarily for the prevention of falls [6-8]. Control of disruptive behaviour, safe use of medical devices and other reasons are also frequently reported [2]. According to international evidence it is questionable, whether physical restraints are effective and safe devices [3,9]. Observational studies rather suggest an association with adverse effects like physical harm, for example serious injuries and increased mortality. Also, social and psychosocial adverse events like reduced psychological wellbeing or decreased mobility have been reported to be associated with physical restraints [3,10].

In the past decades different efforts have been undertaken to reduce the use of physical restraints with older people, starting in the US in the 1980s [11]. Recently, several trials have been conducted mainly evaluating multi-faceted interventions for the reduction of physical restraints, consisting of different components like educational sessions for nurses or information about alternatives [12-17]. The studies did not consistently result in clinically meaningful minimisation of restraints. A Cochrane review on the efficacy of interventions to reduce physical restraints in long-term geriatric care is in preparation [18].

In view of the substantial prevalences of physical restraints in German nursing homes an effective restraint minimisation approach is urgently required. The pronounced centre variations indicate that standard care does not necessarily imply physical restraints. An evidence-based guidance may be a powerful tool to deliver restraint-free care in German nursing homes and to overcome practice variations [19,20]. A systematic search did not reveal publicly available evidence-based guidelines for the avoidance of physical restraints in nursing homes [21]. Thus, we developed a guidance and aim to investigate the efficacy of an intervention programme based on this guidance within a randomised controlled trial.

## Methods

### Study design and setting

The study is a cluster-randomised controlled trial over six months with nursing homes randomised either to the intervention group or to the control group receiving standard information.

### Ethical considerations

The protocol has been approved by the ethics committee of the Hamburg chamber of physicians and the regional data protection office as well as by the ethics committee of the University of Witten/Herdecke. Written informed consent will be obtained from the heads of the participating nursing homes. As requested by the ethics committees and the data protection office, investigators have no direct access to residents' data. All resident-related data will be pseudonymised before given to investigators. During direct observation to assess restraint prevalence, investigators will always be accompanied by a member of the nursing staff. Investigators will enter residents' rooms only after the staff member had asked the resident, if he or she agreed to be visited.

### Study intervention

The methodological framework of the guidance development has been based on internationally suggested approaches like a multidisciplinary guideline development group, methodological training of group members, methodologically sound development of recommendations, an inclusion of resident representatives and relevant stakeholders, an external review and critical appraisal [22-24]. The methodological framework has been published in advance [20]. The guidance comprises 24 statements on relevant interventions to avoid physical restraints.

Taking into account the poor evidence for most interventions, the group mostly made weak recommendations or felt unable to provide any recommendation. A single strong recommendation was made for "educational programmes".

Based on the guidance, an intervention programme has been developed targeting the avoidance of physical restraints. The programme consists of structured information for nursing staff, provision of written information material, and an intensive one-day training workshop for nominated nurses who will be responsible for all issues concerning physical restraints within the centres.

The development of the intervention followed the theory of planned behaviour [25]. A structured single information session of approximately 90 minutes will be provided for each cluster of the intervention group, so that at best all nurses will be informed. The information programme intends to sensitise nurses about the matter of physical restraints and the message of the guidance by addressing their subjective attitudes and experiences. By means of interactive training sequences nurses are motivated to discuss and develop alternative approaches. As supporting materials they receive a short version of the guidance and reminders like posters, pens, mugs, and note pads. Information materials like brochures and flyers for relatives and legal guardians will be provided. The nominated nurses of each cluster will attend a one-day intensive training workshop concerning their role and tasks within the intervention's implementation process. In-depth information and education about the avoidance of physical restraints will be provided.

A declaration will be signed by the representatives of the participating clusters which should be posted in the nursing homes' foyers. Nurses in charge will be announced in this context as contact persons for residents, relatives, physicians, and legal guardians.

Representatives of the control group clusters will receive personal and written brief standard information on legal and scientific evidence on physical restraints and alternatives aimed to avoid measures. No further intervention will be carried out in the control group.

### Identification of clusters and participants

**Figure 1:**
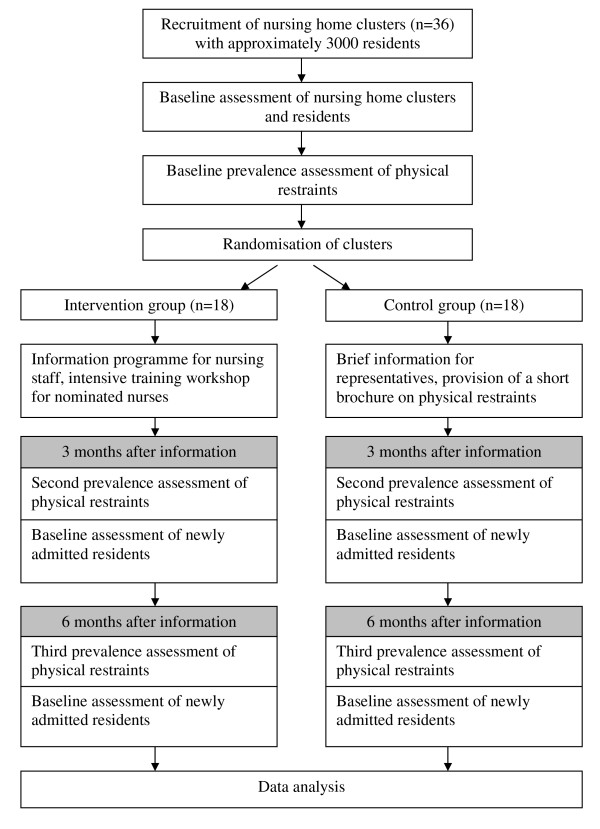
**Summary of trial design**.

Recruitment of nursing homes takes place in the city of Hamburg, Northern Germany, and in surrounding cities of Witten/Herdecke, West Germany. Each nursing home has to fulfil the inclusion criteria of at least 20% self-reported prevalence of physical restraints. A cluster is defined as a nursing home by itself or an independently working unit of a large nursing home, including all residents. Descriptive data on the cluster, participating residents and prevalence data on physical restraint and psychotropic medication will be collected by a nurse supported by an external investigator. Figure 1 shows the summary of the trial design.

### Randomisation

Computer generated randomisation lists will be used for allocation of clusters in blocks of four, six and eight nursing homes. Randomisation will be stratified by region (Hamburg and Witten/Herdecke). Clusters will be allocated after collection of baseline and prevalence data by an external researcher, not involved in the study. The external researcher informs each cluster about its group assignment. Statistical analysis will be conducted after six months at the end of the study to avoid an influence of the investigators by interim results.

### Clinical outcome measures

The primary outcome is the number of residents with at least one physical restraint after six months of follow-up. Physical restraints are defined as any device, material or equipment attached to or near a person's body, which cannot be controlled easily or removed by the person and which deliberately prevents or is deliberately intended to prevent a person's free body movement to a position of choice [26].

At first, characteristics of nursing homes and residents, the prevalence of physical restraints and psychotropic medication will be collected. Data collection instruments used in a previous epidemiological study will be adapted to this study's requirements [4]. Each participating resident will get a code number. Prevalence data of physical restraints will be obtained by direct observation on three occasions on one day (morning, noon, evening) by trained external investigators. Data will be collected before randomisation, after three and after six months. The external investigators collecting the data will be blinded to allocation of nursing homes. The populations assessed at the three data collection time points will slightly vary, since some residents will have terminated the study period ahead of time and other residents will have been admitted between two data collections. For residents admitted to the nursing home between two data collection dates, an abbreviated baseline description will be performed. Reasons for early study termination will be assessed. Secondary outcome measures are the number of falls and fall-related fractures. Nursing staff will document fall events within their in-house documentation system. If no documentation sheet for fall events exists, it will be provided by the researchers.

Cost parameters on the expenses spent for the implementation of the intervention will be collected alongside the trial.

### Process evaluation

Since we aim to implement a complex intervention programme intervening in a complex system, more insight into nurses' comprehension of the restraint reduction approach, the dissemination and delivery of the intervention is needed. Collection of process data will allow us to draw conclusions about potential barriers and facilitators of the intervention [27].

Nurses' knowledge and self-efficacy will be determined at the end of the structured single information session to assess proceeding of the new message. During follow-up, the nominated nurses will be contacted monthly during the first three months by telephone in order to explore barriers and facilitators of the intervention's implementation. Between the three and six month data collection visits, in all intervention group clusters one randomly selected staff nurse will be personally interviewed, whether the restraint reduction approach has been recognised and how it has been perceived.

### Sample size calculation

We expect a significant reduction of the prevalence of physical restraints from 33% to 21%. Assuming comparable conditions as in the epidemiological study [4], a sample size of 2.824 residents in 34 nursing homes with a mean cluster size of 83 residents is required. Presuming a drop-out rate of 5% nursing homes and 2% residents, 36 nursing homes with a mean cluster size of 85 residents need to be recruited [28]. It is planned to recruit 30 nursing homes in Hamburg and six in Witten/Herdecke. The assumed prevalence of 33% in the control group is consistent with the cluster-adjusted estimation of the subpopulation of all nursing homes of the epidemiological study with a prevalence of at least 20% [4]. An intra-class correlation coefficient of ICCC = 0.034 and a design factor of DF = 5.0 were estimated for the primary outcome of physical restraint. Statistical significance is defined as α = 0.05, power is defined as 90%.

### Statistical analysis

The analysis population consists of participants seen at least once during the prevalence data collection at six months. The main outcome is the number of residents with at least one physical restraint after six months and will be analysed by using a two-sided cluster-adjusted chi-square test at a level of significance of α = 0.05 [27]. Baseline data of the three measuring points will be described for the control and intervention group. Secondary outcome measures i.e. incidences of falls and fall-related fractures will be analysed by using cluster-adjusted Poisson-regression models, assuming Poisson-distributions. For the data interpretation the partial data dependency is to be taken into account. The incidence analysis refers to residents of two observation periods: those entering the study at the beginning and after three months of follow-up.

### Time plan

The information programme has been piloted in four nursing homes, who will not participate in the trial. The complete intervention will be piloted in the first two nursing homes allocated to the intervention group. If possible, these will further participate in the trial. Recruitment of clusters started in March 2009 and is expected to be completed in May 2009.

## Discussion

In case the intervention will not succeed, the randomised controlled trial will be followed by a three-month pre-post-study investigating an optimised intervention programme delivered within the control group. Statistical analysis will be planned before starting this study. Results will only be interpreted as explorative.

## Competing interests

The authors declare that they have no competing interests.

## Authors' contributions

GM, IM, and SK were responsible for identifying the research question. GM developed the study protocol and design with significant support of IM and SK. AH developed the one-day training workshop with support of the other authors and was responsible for drafting this paper. All authors commented on the paper drafts and read and approved the final manuscript. AG developed the information programme, supported by the other authors, BH contributed as statistician. All authors read and approved the final version of the manuscript.

## Pre-publication history

The pre-publication history for this paper can be accessed here:


